# Treatment of cardiac nocardiosis in a post-renal transplant patient of systemic nocardiosis

**DOI:** 10.15171/jcvtr.2019.27

**Published:** 2019-05-28

**Authors:** Muddassar Mahboob, Sana Hassan, Ashraf Ali Attia, Ebadur Rahman, Muhammad Shoaib Khan, Syed Rizwan Bokhari

**Affiliations:** ^1^Prince Sultan Military Medical City, KSA; ^2^Jinnah Hospital, Lahore, Pakistan; ^3^Tulane University, New Orleans, USA

**Keywords:** Nocardia, Opportunistic Infections, Cardiac Nocardiosis

## Abstract

In general, nocardia infects immunosuppressed patients, however, sometimes it can also infect immunocompetent individuals. Nocardia infection can disseminate to any organ system of the body but the pulmonary system is the most commonly involved system. In some rare cases, the heart can also be involved and the resulting cardiac mycetoma can be treated successfully with antimicrobials without the need of surgery, unlike fungal cardiac mycetomas wherein surgery may be required in addition to antimicrobial therapy. We present an interesting case of post-renal transplant cardiac nocardiosis, which was treated successfully with a course of antibiotics.

## Introduction

In general, nocardia infects immunosuppressed patients, however, sometimes it can also infect immunocompetent individuals. Nocardia infection can disseminate to any organ system of the body but the pulmonary system is the most commonly involved system. In some rare cases, the heart can also be involved and the resulting cardiac mycetoma can be treated successfully with antimicrobials without the need of surgery, unlike fungal cardiac mycetomas wherein surgery may be required in addition to antimicrobial therapy. We present an interesting case of post-renal transplant cardiac nocardiosis, which was treated successfully with a course of antibiotics.

## Case Report

We report here a forty nine years old male who is a known case of hypertension, diabetes and end-stage renal disease secondary to membranous glomerulonephritis, and who remained in regular hemodialysis for two years before getting a cadaveric transplant. He had an induction with anti thymocyte globulin and was maintained on triple immunosuppressive regimen and his best creatinine post transplant was 1.4 m/dL. After 2 months, he came with productive cough, high grade fever and mild leukocytosis (WBC count of 11.3 x 10^3^). On examination he was stable, conscious and oriented.

**Figure 1:**
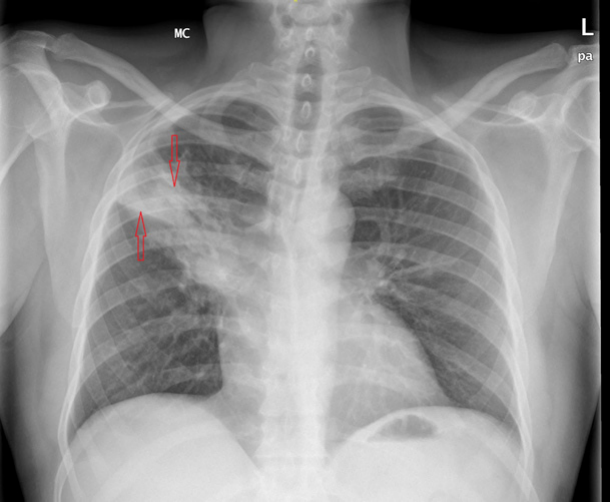
Chest X-ray, postero-anterior view: mass (as pointed by red arrows) seen as consolidation in the upper lobe of the right lung.

**Figure 2:**
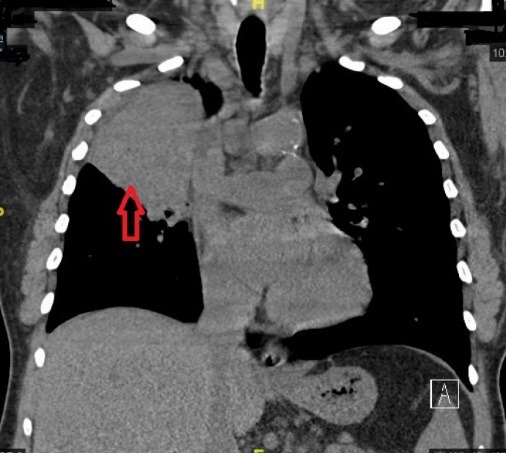
CT Scan Chest without contrast: Red arrow pointing at mass-like consolidation in the right lung.

**Figure 3:**
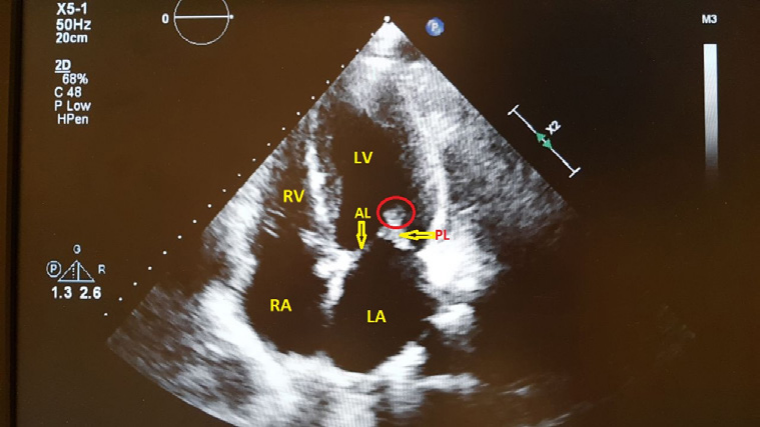
4 Chamber view of the Heart on TTE: Mass (encircled in red) is seen to be attached to the posterior leaflet of the mitral valve.

On chest examination, bronchial breathing was heard and the chest radiograph showed solid consolidations in the right upper lung lobe (Figure 1). Subsequently performed, a high resolution CT chest depicted a mass-like consolidation in right upper lobe (Figure 2) measuring 7cm x 10 cm with air bronchogram. Bronchoscopy was performed and biopsies were taken, which revealed filamentous gram-stain positive bacteria. The sample was negative for Ziehl-Neelsen staining for acid fast bacillus. Polymerase chain reaction (PCR) for tuberculosis (TB) and subsequent culture for TB was negative. On routine TTE, the patient had a mobile mass (0.3 cm x 0.85 cm) attached to the posterior leaflet of mitral valve (Figure 3). This was an incidental finding as pre-transplant TTE was normal. A PCR for Nocardia came out to be positive.

Based on the above mentioned studies, the patient was started on IV TMP-SMX (Trimethoprim- sulfamethoxazole) and Imipenem and this combination was continued for four weeks. Our patient received initial induction therapy with IV TMP-SMX (Trimethoprim- sulfamethoxazole) and Imipenem and this combination was continued for four weeks. The patient showed swift recovery, with frequent radiological imagings showing gradual improvement of lung consolidation. Intracardic mass disappeared after two weeks of treatment. Following the response to antibiotic therapy, the patient was kept on maintenance dose of oral TMP-SMX 960 mg once a day for six months.

## Discussion

To our best knowledge there are claims of cardiac nocardiosis been reported in the past. However, we did not find any exclusive case report solely on cardiac nocardiosis during our extensive literature search online. Cases of the cardiac fungal mycetoma have been demonstrated before. Mycetoma is a chronic infection and is considered as an occupational disease.^1^ Mycetoma infection can be caused by fungi or bacteria and is, respectively, called as mycotic mycetoma and actinomycetoma.

Actinomycetoma is most commonly attributed to nocardia species, particularly Nocardia brasiliensis. Nocardia is an aerobic, gram positive bacillus which appears as branching filaments under the microscope. It is acid fast bacterium, however, its extent of acid fastness is dependant upon the mycolic acid composition in the cell wall and type of stain used.^2^ Nocardia is an opportunistic pathogen and the majority of infections occur in immunocompromised patients,^3^ as is true in our case. However, immunocompetent individuals can also be affected. The disease can spread to almost any organ of the body but the pulmonary system remains the most frequent primary site of involvement.^1^ In recipients of solid organ transplant, nocardia infection has been well described in kidney, heart, and liver recipient.^4^ As inhalation is the primary route of bacterial exposure, pulmonary nocardiosis is the most common clinical presentation of infection.^3^

The chest radiograph can show focal or multifocal disease with lesions ranging from nodular infiltrates, consolidations as well as cavitary lesions.^5,6^ Taking into account, suppressed immune system and radiographic findings, PCR and culture for TB performed subsequently turned out to be negative. However, bronchoscopic biopsies revealed filamentous gram positive bacteria and PCR for Nocardia came out to be positive. 16S rRNA gene analysis was used for speciation. PCR is a rapid and sensitive method for detection of Nocardia in blood and different visceral organs.^7^ In comparison, Nocardia specie usually require 5-21 days for growth in routine aerobic culture.^8,9^ Interestingly, TTE performed as a part of work up showed a mobile mass attached to the posterior leaflet of the mitral valve. TTE performed prior to the renal transplant was unremarkable. Therefore, this new growth on the mitral valve was, at least for the time being, attributed to nocardia infection.

**Figure 4:**
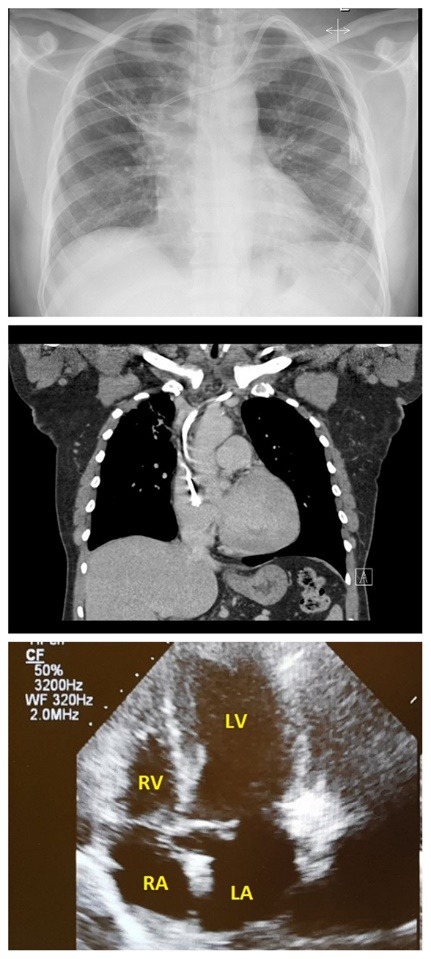
Post-therapy imaging: Mass is consistently seen to have disappeared as seen across three different imaging modalities. Upper Panel; Chest X-Ray Postero-anterior view. Middle Panel; CT Scan Chest with contrast. Lower Panel; 4 chamber view of the Heart on TTE.
LA, left atrium; LV, left ventricle; RA, right atrium; RV, right ventricle; AL, anterior leaflet of mitral valve; PL, posterior leaflet of mitral valve.

Nocardiosis is usually treated with the combination of TMP-SMXas sulfonamides have been the antimicrobials of choice to treat nocardiosis for the past 50 years.^2,10^ Other agents with activity against Nocardia include amikacin, imipenem, meropenem, ceftriaxone, cefotaxime, minocycline, moxifloxacin, linezolid, and amoxicillin-clavulanic acid.^3^ Based on the results of the above mentioned investigations, the patient was started on IV TMP-SMX and imipenem. The idea of combination therapy with imipenem and TMP-SMX, was to provide enhanced bactericial activity.^11^ Over next two to four weeks, the patient showed marked improvement of clinical status, radiographic findings, and TTE demonstrated cardiac mass disappeared completely (Figure 4), affirming our assumption of cardiac nocardiosis. Hence, we can surmise that cardiac actinomycetoma can be treated (at least initially) with medications. On the other hand fungal mycetoma usually requires both prolonged antifungal medication and surgery.^12^

## Conclusion

Cardiac nocardiosis is a rare pathology, which sometimes can inflict immunosuppressed patients. It can be cured medically with antimicrobials and surgical intervention is usually not required. In contrast, fungal mycetoma usually requires surgical intervention in addition to antimicrobials.

## Ethical Approval

In this case study, patient confidentiality has been strictly preserved.

## Competing interests

There was no conflict of interest.

## Funding

This study received no grant from any funding agency in the public, commercial or not-for-profit sectors.

## Acknowledgements

We wish to thank Syed Ziad Ali (FCPS) for editing the article.
